# Applying Health Systems Science Competencies to Contribute to the COVID-19 Pandemic Response

**DOI:** 10.7759/cureus.74154

**Published:** 2024-11-21

**Authors:** Victoria S Elliott, Resney Gugwor, Jonathan Y Siden, Maya M Hammoud, Kevin Heckman, Kimberly D Lomis

**Affiliations:** 1 Medical Education Outcomes, American Medical Association, Chicago, USA; 2 Obstetrics and Gynecology, Brigham and Women’s Hospital, Boston, USA; 3 Obstetrics and Gynecology, Massachusetts General Hospital, Boston, USA; 4 Obstetrics and Gynecology, Harvard Medical School, Boston, USA; 5 Obstetrics and Gynecology, University of Michigan Medical School, Ann Arbor, USA

**Keywords:** competency frameworks, educational competencies, health care delivery science, health systems science, interprofessional education, leadership training, pandemic response, teamwork, technology, undergraduate medical education

## Abstract

In the first quarter of 2020, nearly all U.S. medical schools transitioned to virtual instruction and removed medical students from clinical settings because of the emerging COVID-19 pandemic. While medical school education continued in a different form, questions emerged about the effectiveness of instruction during this hopefully once-in-a-lifetime public health crisis. Many medical students involved themselves in the pandemic response either by creating projects that addressed emerging needs or joining projects created for them by faculty or school leadership. Prior research has indicated that medical student involvement in these projects had educational value, although the nature of that value has not been articulated in the context of existing competency frameworks.

The authors reviewed 55 abstracts focused on student-led pandemic-related projects entered into the 2020 American Medical Association (AMA) Accelerating Change in Medical Education Consortium Health Systems Science Student, Resident, and Fellow Impact Challenge and identified which health systems science (HSS) competencies students most likely acquired through their participation in these projects. The authors conclude that these medical students leveraged these experiences to strengthen key HSS skills, especially in teaming, leadership, and technology, and they advanced their professional identity formation as physicians despite significant disruption to their training. This crisis was an unmatched opportunity for exploring core HSS concepts, and medical students developed meaningful competencies by alternate means. Although medical students in medical school in 2020 and 2021 may have gaps in some areas that need to be addressed, the authors posit that those who engaged in these projects gained strengths that they would not have otherwise acquired.

## Introduction

When the COVID-19 pandemic emerged in the first quarter of 2020, the Association of American Medical Colleges (AAMC) called for medical schools to temporarily suspend clinical rotations for medical students [1], and nearly all medical school instruction became virtual [2]. Despite being sidelined from clinical roles, many medical students used the flexibility of virtual learning to pursue projects addressing the impact of the COVID-19 pandemic and the pandemic response [3-5]. Questions have surfaced about the effectiveness of medical education during this public health crisis [6]. The passing rate for the United States Medical Licensing Exam® (USMLE®) held steady in 2020 and 2021 [7], but some studies suggest that, although knowledge transfer during virtual learning was equivalent to in-person education, the transfer of skills and attitudes was not [8,9].

Medical students who completed clinical clerkships in 2020 may have gaps in their education because of the pauses and/or modifications implemented as the COVID-19 pandemic emerged and progressed. Conversely, the pandemic may have opened opportunities for innovation in increasingly important areas of medical education, such as health systems science (HSS), which is defined as a “foundational framework for the study and understanding of how care is delivered, how health professionals work together to deliver that care, and how the health system can improve patient care and health care delivery” [10]. HSS is becoming a common core component of medical education [11] but remains difficult to integrate into the clinical curriculum [12]. We suggest that students educated in HSS leverage and strengthen their skills to contribute to the COVID-19 pandemic response, demonstrating continued professional identity formation during this challenging time. This public health crisis, which illustrated the interconnected nature of health systems, physicians, patients, and patient health outcomes, provided an experiential format and urgency for the direct application of core HSS concepts. Many see clerkships as focused on a specific discipline, but much of what is being fostered is broader competencies [13]. Students may have lost some opportunities to develop discipline-specific competencies, but this review indicates participation in the pandemic response facilitated the continued development of broader competencies.

Prior analysis indicates most of the tasks undertaken by medical students during the COVID-19 pandemic had educational value [14]. Consequently, we ask, what did medical students learn from the value-added roles they created, or the roles created for them, in this public health crisis?

We acknowledge that graduate medical education was also significantly changed during the early months of the COVID-19 pandemic, but those changes are beyond the scope of this paper. While residents were redeployed and may have had clinical roles and educational experiences that differed from those initially planned or expected, residents were not removed from direct patient care.

## Materials and methods

We reviewed the medical student-led projects focused on COVID-19 entered into the 2020 American Medical Association (AMA) Accelerating Change in Medical Education Consortium Health Systems Science Student, Resident, and Fellow Impact Challenge to identify relevant HSS competencies [15]. These projects act as a convenience sample that we linked with HSS competencies included in the AAMC Core Entrustable Professional Activities (EPAs) for entering residency, USMLE physician tasks and competencies, and USMLE content outline (step 1) [16]. We used the AAMC Curricular Developers Guide to identify key competencies associated with the Core EPAs [17]. If project abstracts reported the number of students involved, we included that as part of our analysis. If no number was reported, we only included the number of medical student authors in the count of impacted medical students.

One author on this paper (VSE) reviewed each abstract three times, looking for keywords relevant to the respective educational domains, utilizing criteria developed with the full author team on this paper, and judgment as to whether a particular project could lead to competency in one or more of the 18 HSS domains included in the AAMC Core EPAs for entering residency, USMLE physician tasks and competencies, and USMLE content outline (step 1) [16].

## Results

Teams of medical students, residents, fellows, and their faculty mentors submitted 75 abstracts to the 2020 AMA Accelerating Change in Medical Education Consortium Health Systems Science Student, Resident, and Fellow Impact Challenge. Twenty of the abstracts were eliminated from this review because those projects were led by residents or fellows. This left 55 abstracts, and a minimum of 3,426 medical students were involved, participated, or otherwise known to be affected by these projects. 

The results of this review are in Table 1. A notable percentage of projects were shared or disseminated in some way with other institutions, demonstrating interest from the broader medical education community. Another aspect of these projects highlighting their value is that all of them had a faculty mentor, and several had significant faculty involvement. This is an indication that, even at a time when many faculty were overwhelmed with clinical, administrative, crisis management, and education duties, there remained a strong interest in creating meaningful learning opportunities, especially in HSS. Faculty engagement is key to supporting such responsive learning opportunities in the future.

**Table 1:** Characteristics of submitted abstracts Teams of medical students, residents, fellows, and their faculty mentors entered 75 abstracts into the 2020 American Medical Association Accelerating Change in Medical Education Consortium Health Systems Science Student, Resident, and Fellow Impact Challenge. This review is limited to the 55 abstracts written by teams led by medical students.

Characteristics	Yes	No
The abstract reported the number of students involved.	25 (45%)	30 (55%)
The abstract reported that the project was disseminated in some way.	16 (29%)	39 (71%)
The project included significant faculty involvement.	14 (25%)	41 (75%)
A faculty member actively collaborated with medical students.	8 (14%)	47 (86%)
The idea for the project came from a faculty member.	6 (11%)	49 (89%)

**Figure 1:**
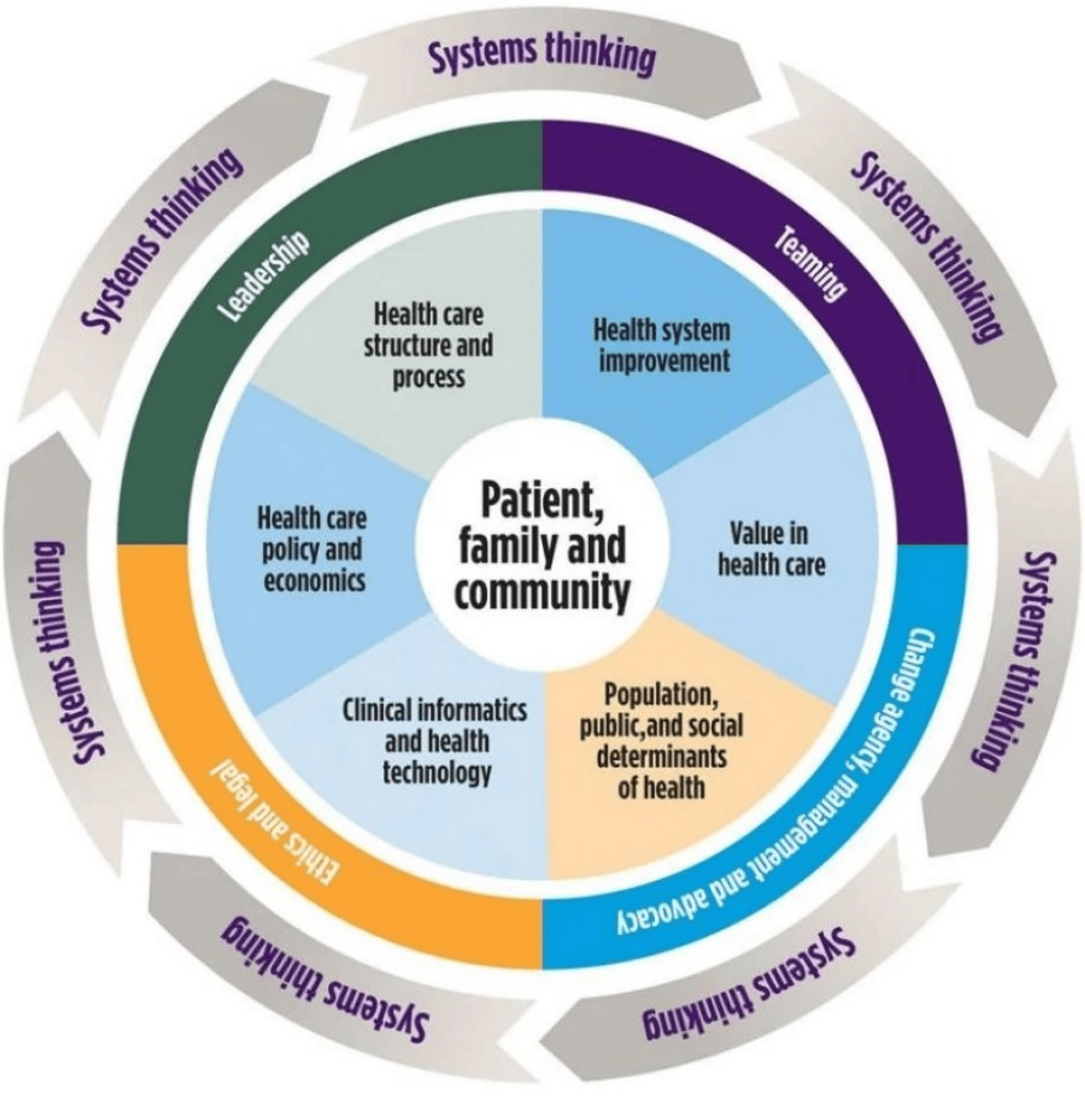
The core functional, foundational, and linking domains of health systems science Copyright American Medical Association. Used with permission.

All projects addressed at least three domains of competency. Projects addressed a mean of seven HSS domains. This validates the premise of the HSS framework: physicians need foundational understanding across all of these interconnected domains to address the needs of patients and communities, as indicated in Figure 1 [10].

**Figure 2:**
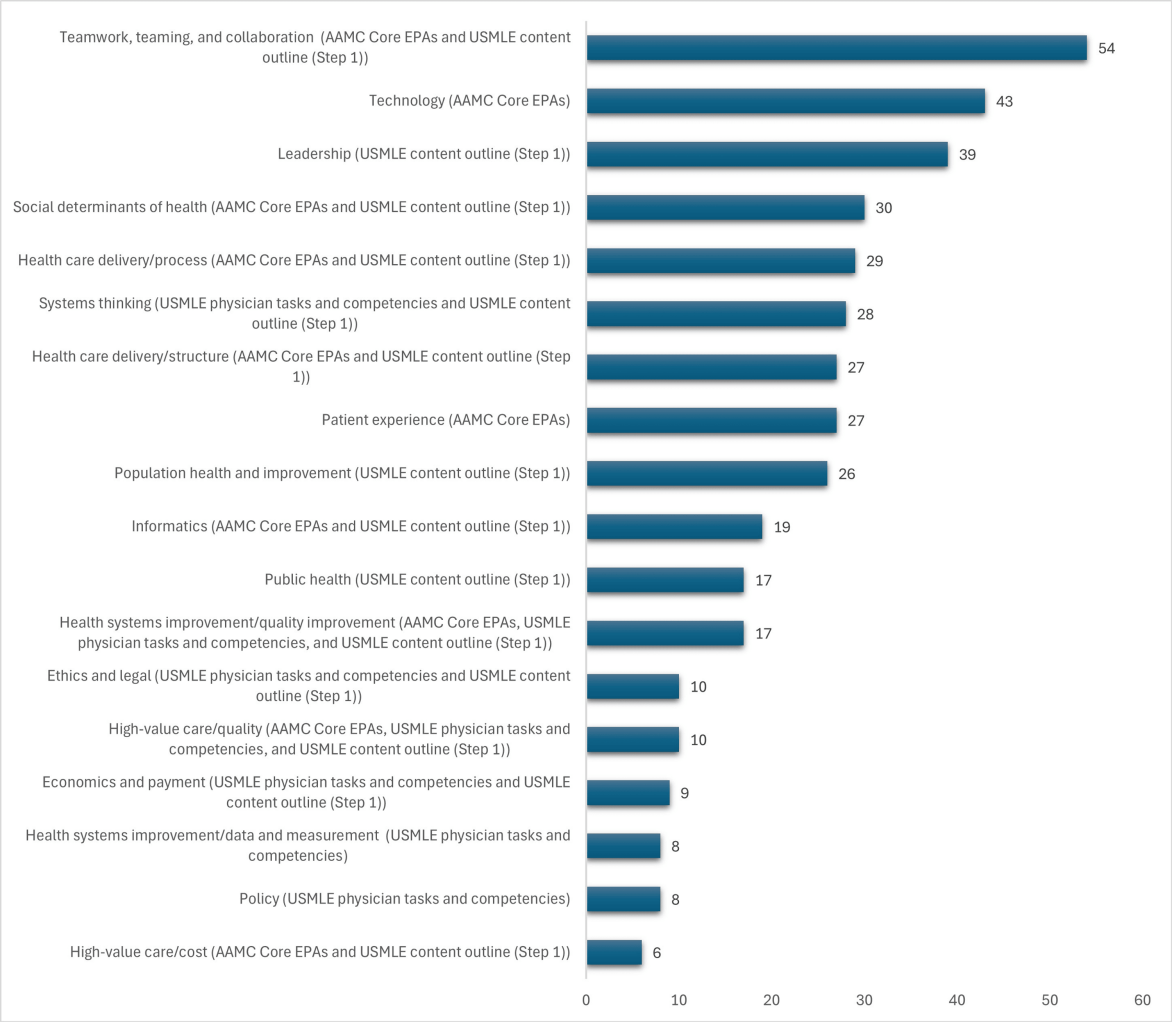
Number of abstracts incorporating each domain of HSS All projects addressed at least three domains of competency. Projects addressed a mean of seven HSS domains. HSS, health systems science

The most common domains addressed in these projects were teaming (corresponding to both AAMC Core EPAs and USMLE content outline (step 1)), leadership (corresponding to USMLE content outline (step 1)), and using information technology to optimize learning and care (corresponding to AAMC Core EPAs). A full breakdown is included in Figure 2.

## Discussion

Learning to work as part of an interprofessional team

The team of first-year medical students from the School of Medicine, University of California, Irvine, CA, in the personal impact statement accompanying their abstract, quoted: “We will carry the knowledge we’ve gained and the sense of responsibility for others throughout our personal and professional lives as we remember the sacrifices made by those in our community, many of whom will also one day be our patients and colleagues.”

Educating medical students to become physicians who lead and work as part of interprofessional teams has long been recognized as important yet challenging [18], and teaming, especially with other healthcare professionals, is a particularly difficult skill to promote through virtual teaching modalities [19]. When considering whether a project could contribute to competency in this domain, we eliminated abstracts with one author working primarily with a mentor and without other teammates, either cited as co-authors or mentioned in the text of the abstract. A project was considered as potentially leading to teaming competency if it required the project leader to work and collaborate with other medical students, health professions students, or health professionals. A total of 54 of the 55 projects (98%) would likely contribute to developing competency in teaming, and 38 of 55 (69%) were explicitly interprofessional. Projects were considered interprofessional if the abstract stated that the project was interprofessional in nature or named other health professionals or health professions students, including dentists, medical assistants, public health practitioners, nurses, and laboratory technicians.

For example, the team of first-year medical students at the University of California, Irvine, quoted at the opening of this section, collaborated with their institution’s procurement and contracting department to identify the necessary types of personal protective equipment (PPE), set up a system for processing donations, recruit volunteers, and set up donation drop-off locations, storage solutions, and decontamination procedures. They modeled their PPE drive on those run by medical students at other institutions, worked with their institution’s public relations department to disseminate information about needs, and, over four weeks, collected more than 31,000 pieces of PPE for their institution. The medical students acted as a bridge between overwhelmed healthcare workers and community members who donated standard PPE from their own supplies or used their skills and fabric stash to sew masks. The students, in their own words, “gained an enhanced understanding of the myriad of roles within different hospital departments and essential employees required to keep both our health care providers and communities healthy and safe.”

Other projects addressing the teaming domain included one at New York University Grossman School of Medicine, New York, NY. That institution dealt with a surge of patients in the first half of 2020, but families and other visitors were barred from entering facilities. This made communication between physicians and families especially challenging. The mentor of this project, Katherine Hochman, MD, recruited 115 medical students to become part of NYU Family Connect. Dr. Hochman paired these students with physicians who mentored and trained them to remotely gather clinical information from patient charts, participate in virtual rounds, provide daily clinical updates to family members, and document these interactions. A total of 68 of 115 (59%) volunteers completed a survey about their experiences. Of the respondents, 60 of the 68 (88%) believed that the program was a critical part of their educational experience. A total of 59 (87%) said the program increased their ability to work with a diverse team of healthcare professionals, and 48 (70%) stated that their ability to communicate medical knowledge with physicians who were not directly supervising them improved.

Becoming a leader

A team of medical students at the University of California, San Francisco, CA, in the personal impact statement accompanying their abstract, quoted: “For us as students specifically, this pandemic…represented an unprecedented opportunity to step up in non-traditional roles, to contribute to the health care system, and gain invaluable preparation as future health care leaders.”

Leadership is another competency medical education has long struggled to impart to medical students despite growing recognition of its importance [20]. We linked a project to leadership competency if the authors of the abstract assembled groups of people to complete a task or stated that they felt they gained leadership, change agency, and/or advocacy skills, and 39 of 55 (71%) of projects fulfilled these criteria.

The team at the University of California, San Francisco, quoted at the beginning of this section, responded to the blood supply shortage triggered by the COVID-19 pandemic by bringing together hospital leaders, blood supply managers, educators, and students. They formed a working group to create and implement a model for safe blood donation, maintained their institution’s blood supplies, and created a unique medical student educational experience in crisis response.

Other projects that informed and strengthened medical student leadership skills included one at the University of Tennessee Health Science Center, Memphis, TN. When the institution removed medical students from clinical rotations, medical student leaders worked with senior administration to plan, launch, and operate a drive-thru COVID-19 testing site for the community. Students, in collaboration with faculty mentors and city officials, developed all training, testing, and safety protocols. Over a six-week period, five third- and fourth-year medical students led the project with the support of more than 170 medical, nursing, and dental student volunteers. Funded by the Memphis mayor’s office, this testing site quickly became the region’s largest and was soon followed by a second site. Before transitioning to permanent staff, students tested over 3,600 individuals.

Utilizing technology

Josh Gray, MS2 in 2020, Johns Hopkins University School of Medicine, Baltimore, MD, in the personal impact statement accompanying his abstract, quoted: “My initial dismay about not being able to contribute to the cause has been transformed into the opportunity of a lifetime.”

Technology has become more important than ever during the COVID-19 pandemic. People became more reliant on smartphones and computers to meet the most basic human needs. To categorize a project as having the potential to lead to competency in using information technology to optimize learning and care, we required students to actively engage with technology, improve how it was used, or improve the technology itself.

For some technically adept medical students, the pandemic drew on their skills, amplified them, and improved them. Josh Gray, MS2 in 2020 at Johns Hopkins University School of Medicine and quoted at the beginning of this section, created software that automated how a chart abstraction project was managed. This was essential at the start of the pandemic because numerous COVID-19-related questions could only be answered by the data from the unstructured parts of electronic health records (EHR), such as clinical notes. This software trained and onboarded chart abstractors across the health system, assigned chart abstraction tasks, and monitored progress and quality.

Another project that strengthened medical students’ technology skills addressed the ongoing care needs of those who did not have COVID-19 but still required services from a stressed and overtaxed health system. Medical students at the University of Michigan, Ann Arbor, MI (including one of the authors on this paper, JYS) established the COVID-19 Prenatal Support Project. This initiative was designed to guide prenatal care adaptations during the COVID-19 pandemic, including a reduced visit schedule and greater use of telehealth. A total of 55 medical student volunteers, midwives, and physicians from obstetrics and gynecology, maternal-fetal medicine, and family medicine created a prenatal care model using HSS principles. This model integrated various departments, social and institutional resources, and policy changes to ensure patient and community safety while upholding quality care. Organizers enlisted student volunteers to contact over 1,500 patients within 10 days. Student leaders established an internal student-led translation service for non-English-speaking patients, connected patients to community and educational resources, and facilitated social work referrals for mental health and essential resource needs. Additionally, by virtually training medical students to educate patients about telehealth, prenatal care, at-home blood pressure monitoring, and EHR documentation, the project not only reduced risks of viral exposure but also enhanced students’ broader competencies alongside discipline-specific skills.

Limitations

Prerak Juthani, MS3 in 2020 at Yale School of Medicine, New Haven, CT, in the personal impact statement accompanying his abstract, quoted: “This work has made me realize that I am never too small to make a difference.”

This review is limited to the information included in the abstracts submitted to the 2020 AMA Accelerating Change in Medical Education Consortium Health Systems Science Student, Resident, and Fellow Impact Challenge, and reported data varied widely. Notably, while we believe these projects led to HSS competencies, none of the abstracts included an assessment to determine if an HSS-related competency was achieved. Many abstracts included data documenting the magnitude of the project’s impact on patients, other important medical competencies, and the COVID-19 pandemic but lacked the assessment that is frequently a part of designing value-added roles for medical students [21]. This was undoubtedly the result of the COVID-19 pandemic crisis, which made it more important to implement projects such as those reviewed in this article than assess their educational impact. Including assessment in future educational experiences of this kind will be a critical part of confirming their value.

Additionally, these projects involved and impacted at least 3,426 students, a significant number but a small proportion of the nearly 100,000 medical students in the United States [22]. There are obviously many medical student projects implemented in the United States that are not included in our sample. Some medical students did not or could not participate in the pandemic response or the projects that emerged when they were removed from clinical rotations. This review also does not include projects created and led by residents and fellows, which is beyond the scope of this paper.

## Conclusions

We believe our review of this group of abstracts reflects positive professional identity formation among students who took the initiative to contribute to the response in a way that was appropriate to their skill level and available information and resources. This review provides insight into what engaged and involved medical students learned during their work during the initial months of the COVID-19 pandemic and suggests effective strategies for teaching HSS. Student-led and faculty-supported initiatives during the COVID-19 pandemic naturally integrated HSS competencies, and there was a real hunger to develop these skills, although it is interesting to highlight some of the domains that appeared less frequently in these projects. Notably, policy, which is part of USMLE physician tasks and competencies, and public health, which is part of the USMLE content outline (step 1), were some of the less common competencies addressed, but some of the most important aspects of the pandemic response. These areas may need more deliberate effort to generate meaningful roles for learners.

The COVID-19 pandemic changed all of us and impacted us in ways that have yet to become completely clear. We work and live in vastly different ways than we did before. Those who experienced medical school during the peak pandemic years will have different strengths and weaknesses that must be considered as they progress through the medical education continuum.
